# Ab Initio Study of Octane Moiety Adsorption on H- and Cl-Functionalized Silicon Nanowires

**DOI:** 10.3390/nano12091590

**Published:** 2022-05-07

**Authors:** Barbara Ferrucci, Francesco Buonocore, Simone Giusepponi, Awad Shalabny, Muhammad Y. Bashouti, Massimo Celino

**Affiliations:** 1 Fusion and Technology for Nuclear Safety and Security Department, Italian National Agency for New Technologies, Energy and Sustainable Economic Development (ENEA), Bologna Research Centre, 00129 Bologna, Italy; 2 Energy Technologies and Renewable Sources Department, Italian National Agency for New Technologies, Energy and Sustainable Economic Development (ENEA), Casaccia Research Centre, 00123 Rome, Italy; simone.giusepponi@enea.it (S.G.); massimo.celino@enea.it (M.C.); 3 Department of Solar Energy and Environmental Physics, Swiss Institute for Dryland Environmental and Energy Research, J. Blaustein Institutes for Desert Research, Ben-Gurion University of the Negev, Midreshset Ben-Gurion, Building 26, Be’er Sheva 8499000, Israel; awads@post.bgu.ac.il (A.S.); bashouti@bgu.ac.il (M.Y.B.)

**Keywords:** density functional theory, silicon nanowire, passivation, charge density, octane moiety adsorption, electronic structure, band gap

## Abstract

Using first-principles calculations based on density functional theory, we investigated the effects of surface functionalization on the energetic and electronic properties of hydrogenated and chlorinated silicon nanowires oriented along the <112> direction. We show that the band structure is strongly influenced by the diameter of the nanowire, while substantial variations in the formation energy are observed by changing the passivation species. We modeled an octane moiety absorption on the (111) and (110) surface of the silicon nanowire to address the effects on the electronic structure of the chlorinated and hydrogenated systems. We found that the moiety does not substantially affect the electronic properties of the investigated systems. Indeed, the states localized on the molecules are embedded into the valence and conduction bands, with no generation of intragap energy levels and moderated change in the band gap. Therefore, Si-C bonds can enhance protection of the hydrogenated and chlorinated nanowire surfaces against oxidation without substantial modification of the electronic properties. However, we calculated a significant charge transfer from the silicon nanowires to the octane moiety.

## 1. Introduction

Silicon nanowires (SiNWs) are being studied intensively because of their fascinating properties and potential applications in a wide range of areas, such as electronics [1], energy [2,3,4], and chemical and biochemical sensing [5,6]. Thanks to their small diameter of less than 100 nm, the SiNWs have a large surface-to-volume ratio that makes surface effects dominant. This gives them a high sensitivity in chemical surface processes through which it is possible to alter their surface potential and charge distribution [7,8]. Indeed, surface functionalization is one of the best techniques to modify the electronic properties of SiNWs, which can strongly depend upon the bond strength and relative electronegativity of passivating elements and/or molecules [9,10]. On the other hand, the low dimensionality of NWs leads to quantum confinement effects, which can be harnessed to control their electronic properties by varying their diameter and orientation [7,11].

In the field of electronics, photovoltaics and biosensors, an important step in designing SiNW-based devices consists of controlling the immobilization of organic molecules on SiNWs through the surface functionalization [12,13,14]. To this end, a promising approach to control the electronic properties of silicon surfaces is to use monolayers that introduce a net electrical dipole perpendicular to the surface/interface [15,16]. In this way, the work function and the electronic affinity at the surface are modified, and the band offset and band bending at the interface can be tuned, i.e., controlling charge transfer. The band alignment between the hybrid Si and the interface has an important role for the charge transfer for energy devices such as batteries and storage [17]. A typical chemistry approach is to link moieties to the oxidized SiNWs, generally through -OH chemistry. Many pertinent applications have been illustrated, such as the enhancement of nanosensors for biological and chemical species detection by modifying the surface properties with functional groups, as reviewed by Ahoulou et al. [18]. However, for many specific device settings, the presence of native oxide at the SiNW surfaces, which instantaneously forms on the surfaces when exposed to air, induces uncontrolled interface states in the band gap of silicon, degrading the device performance [19]. These effects become more relevant as the diameter of the SiNWs decreases. Because of this, protection of the SiNW surfaces against oxidation is required. Therefore, it is necessary to explore models and methods to predict and control the surface physical characteristics of oxide-free SiNWs and to investigate the geometrical effects of organic molecule adsorption. For example, HF-etched SiNWs show no oxide shell with surface-bound hydrogen atoms (Si-H) that terminate dangling bonds. Nevertheless, exposure of these surfaces (i.e., Si-H) to ambient air causes a rapid oxidation, leading to higher surface recombination velocities [20]. Thus, it is essential to prevent extensive SiNW surface oxidation while preserving the low surface recombination velocities for a longer time. It has been proven that the adsorption of organic molecules to the Si surface through Si-C bonds allows a lower rate of oxidation and electron–hole recombination than H-terminated Si surfaces [12]. To this end, the H-terminated Si surfaces can be alkylated using chlorination/alkylation processes.

In the present paper, the energetics of SiNWs as well as the dependence of the electronic properties on diameter have been investigated based on first-principles calculations. The dependence on passivating species has been examined by comparing the effect of the total surface passivation with (H-) hydrogen, and (Cl-) chloride atoms for three different diameters of SiNWs oriented along the <112> direction. This investigation drives the choice of the SiNW model to be used for the analysis of the adsorption of the octane moiety on the (111) and (110) surfaces of both the H- and Cl-fully passivated SiNWs. The ab initio approach was established to find the optimized geometry and calculate the formation energy of the systems.

## 2. Methods

We performed first principles total energy calculations to study the electronic properties of different silicon-based nanostructures. All numerical calculations were carried out by using the periodic density functional theory (DFT) as implemented in the PWscf (Plane-Wave Self-Consistent Field) code of the Quantum Espresso package [21]. The exchange-correlation energies were treated with the Perdew, Burke and Enzerholf (PBE) functional [22]. Electron–ion interactions were described by the ultra-soft Vanderbilt pseudopotentials [23]. The geometries of all structures were fully optimized using an energy cutoff of 40 Ry, and a charge density of 400 Ry. The force and energy threshold have been imposed equal to 1.0 × 10^−4^ Ry/Bohr and 1.0 × 10^−5^ Ry, respectively.

Self-consistent calculations to sample the Brillouin zone were performed to investigate the convergence of total energy with respect to different k-point grids. After checking the total energy (*E_T_*) convergence, we chose to use the 1 × 1 × 8 k-point grid. Löwdin analysis was used to quantitatively estimate the charge transfer between the fully passivated SiNWs and the adsorbed octane. The non-self-consistent calculation of the electronic states was performed on a 1 × 1 × 24 k-point grid, which was found to yield a sufficiently accurate representation of the density of states. The band structure has been calculated along a path of 20 points parallel to the kz axis of the Brillouin zone.

Two sets of passivated systems have been considered: silicon nanowires fully passivated with hydrogen atoms (H-SiNWs), and silicon nanowires fully passivated with chlorine atoms (Cl-SiNWs).

Genetic algorithm studies based on DFT and molecular dynamics (MD) calculations have shown that the Si(112) NWs expose the (111) and (110) facets, as reported by Lu et al. [24], in excellent agreement with the HRTEM (High-Resolution Transmission Electron Microscope) characterization [25]. Therefore, each structure was modeled using a supercell periodic along the <112> direction, with a rectangular cross-sectional area enclosed with the (111) and (110) facet. The diameter of each wire was calculated by [16]:

(1) $$d=2\sqrt{A/\pi },$$

where *A* is the SiNW rectangular cross-section perpendicular to <112> direction.

**Figure 1:**
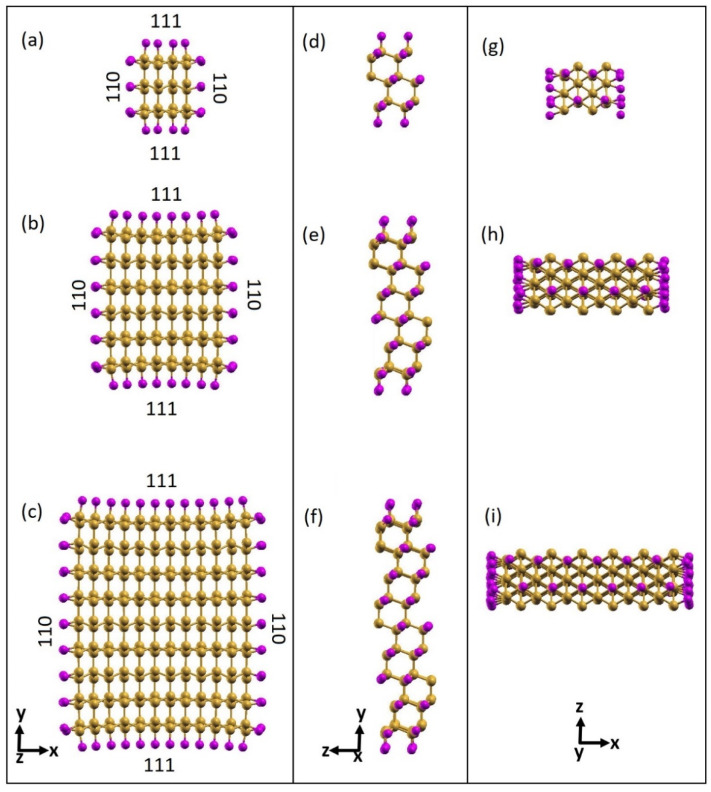
Optimized atomic structures of fully passivated Cl-SiNWs (magenta color: Cl atoms; mustard color: Si atoms). (**a**,**d**,**g**) Si_216_Cl_60_ (*d* = 2.64 nm); (**b**,**e**,**h**) Si_96_Cl_40_ (*d* = 1.671 nm); and (**c**,**f**,**i**) Si_24_Cl_20_ (*d* = 0.71 nm). Left column: cross sections of the <112> Cl-SiNWs enclosed with (111) and (110) facet; central column: side view of the <112> Cl-SiNWs showing the top of the (110) surface; right column: side view of the <112> Cl-SiNWs showing the top of the (111) surface.

For both the passivating species, H and Cl, we modeled three SiNWs labeled as small (*d* = 0.7 nm), medium (*d* = 1.7 nm) and large (*d* = 2.6 nm), consisting of 24, 96, and 216 atoms of Si, and 20, 40, and 60 atoms of Cl (Figure 1) or H, respectively.

## 3. Results and Discussion

### 3.1. Electronic Properties of Fully Passivated Cl- and H-SiNWs

First, we investigated the preferential adsorption sites of single H and Cl atoms on the Si (111) and (110) surfaces. By modeling an eight-layer silicon slab consisting of symmetric slabs at the optimized bulk lattice constant of 5.469 Å, we found that the top site allows the most energetically stable conditions. Based on these results, we computed the formation energy and the electronic properties of the small, medium, and large H- and Cl-SiNWs with H and Cl atoms, respectively, adsorbed on the top sites of the (111) and (110) surfaces.

The formation energy per atom ($E_{F})$ was calculated according to the formula:

(2) $$E_{F}=(E_{T}-\sum_{i=Si,H,Cl}n_{i}E_{i})/n,$$

where $E_{T}$ is the total energy of the full system; $n_{i}$ and $E_{i}$ are respectively the number and energy per atom of each atomic species [26]; and n is the total number of atoms. The energy for Si is taken from the bulk silicon, while the energy for Cl and H are taken from their molecular state. The module of formation energy says how stable or unstable the system is. Lower formation energies (i.e., more negative values) correspond to more stable systems.

The results are reported in Table 1, where we have used the expression Si_n_X_m_ (n, m: number of atoms; X: passivating species) to indicate the reference NW structure. The formation energy per atom of Cl-SiNWs is less than that of H-SiNWs. This means that the Cl atoms better improve the structural stability of the wire with respect to the H atoms. It should be noted that in the two cases, the stability trend is different with respect to the wire dimension. Indeed, moving from the small to the large diameter, the stability increases for the hydrogenated system and decreases for the chlorinated one. This may be ascribed to the different electronegativity of Cl and H atoms, which are 3.16 and 2.1, respectively [27].

**Figure 2:**
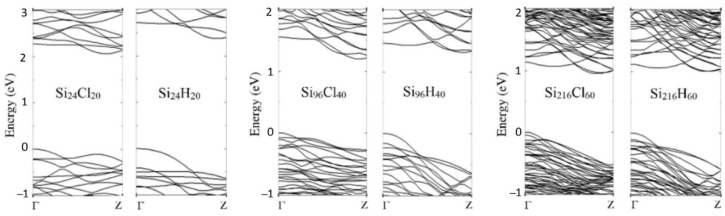
Band structures of fully passivated Cl- and H-functionalized <112> oriented SiNWs. The top of the valence band is set to zero.

**Figure 3:**
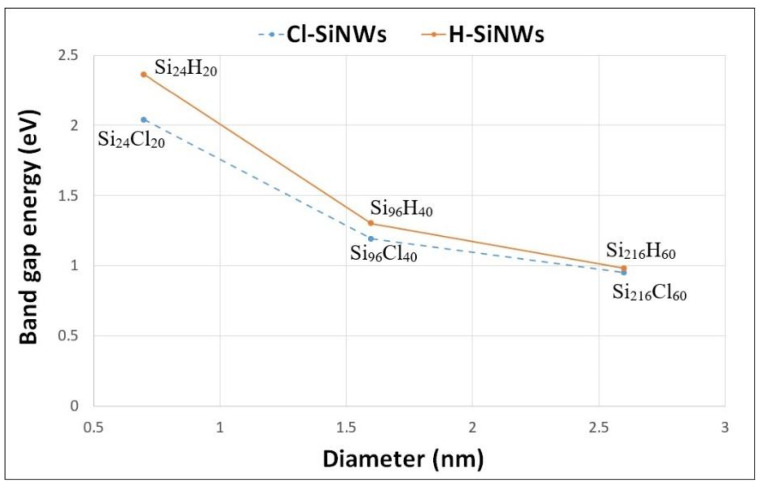
Computed band gap as a function of the diameter of fully passivated Cl- and H-functionalized <112> oriented SiNWs.

Figure 2 shows the band structure of the computed H- and Cl-SiNWs. The valence–conduction band-energy gaps (*E_g_*) are reported in Table 2. It should be noted that the band-gap energy of the three H-SiNWs is larger than that of the respective Cl-SiNWs. These results agree with those reported in reference [9], where the authors observe that the halogenation of 1.5 nm-diameter SiNWs slightly reduces the energy gap with respect to the hydrogenated case. This can be explained because of the higher electronegativity of Cl that leads to a narrower energy gap of the system [7]. For both the hydrogenated and chlorinated systems, due to the quantum confinement effect, the band gap decreases as the SiNWs diameter increases (Figure 3) [28].

The overall features of the band structure for the wires investigated are quite similar. For each configuration, regardless of the passivating species, the maximum of the valence band is located at the Γ point, and the minimum of the conduction band is near the Z point, leading to an indirect band gap (Figure 2). Although the energy gap depends on the passivation species and the diameter of the wire, the gap remains indirect according to the wire orientation [29,30]. Moreover, the difference between the band gap of H- and Cl-functionalized SiNW of large diameter decreases to 0.03 eV.

**Figure 4:**
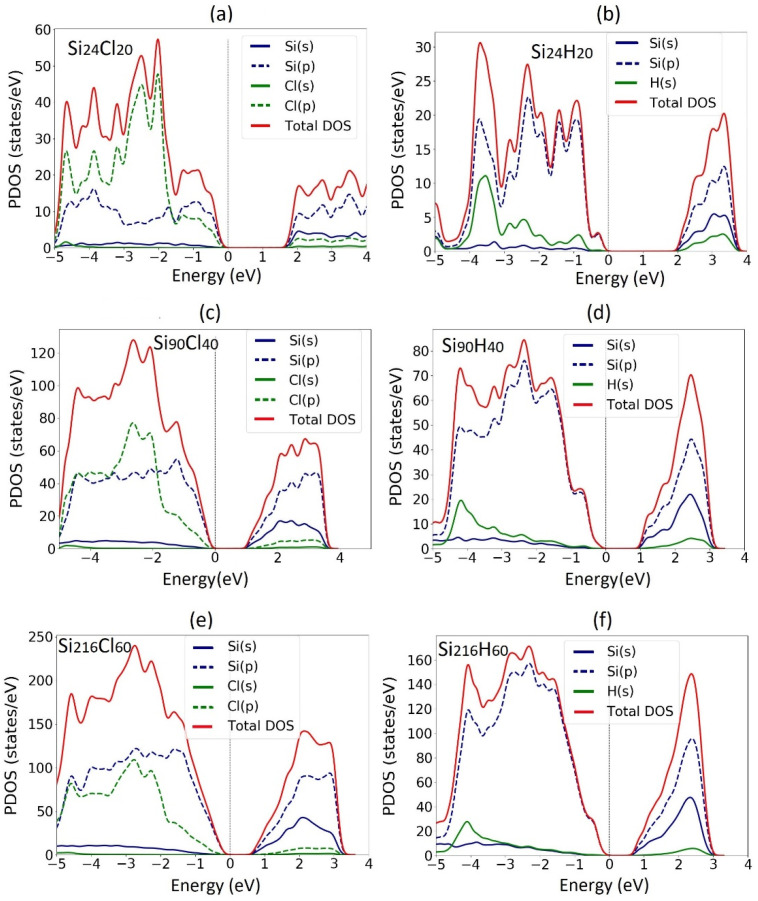
Projected electron density of states (PDOS) of fully Cl- and H- functionalized <112> oriented SiNWs: (**a**,**b**) small; (**c**,**d**) medium; (**e**,**f**) large SiNW.

The calculated partial density of states (PDOS) are shown in Figure 4. The top of the valence band was set to 0 eV in all the plots. From the PDOS analysis, it can be seen that the valence and conduction band edges of the three H-SiNWs are mainly composed of the silicon-p orbitals, Si(p). For the Cl-SiNWs, there is a major contribution of chlorine p-orbitals, Cl(p), due to the interaction of the hybridization of Cl(3p) and surface Si(3p) states [19]. This effect decreases as the diameter increases, due to the relative reduction in the ratio of Cl/Si number of atoms in the wire. Therefore, the medium diameter SiNW has been chosen for the investigation of the molecule adsorption.

#### Differential Charge Density of Cl- and H-SiNWs

**Figure 5:**
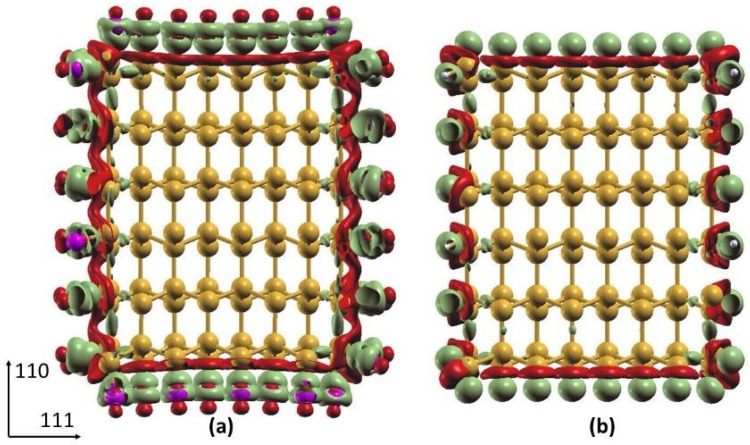
Isosurface of differential charge density of (**a**) Cl-SiNW and (**b**) H-SiNW (mustard color: Si atoms; white: H atoms; magenta: Cl atoms). Green and red regions indicate charge accumulation and depletion, respectively. The isosurface value is set to 0.005.

Taking as reference the medium diameter nanowires, as defined above, we calculated the differential charge density of both H- and Cl-SiNWs to carefully compare their charge distribution at the Si-H/Cl bonds. The differential charge density has been calculated as the difference of the charge density of the full structure and the charge density of the pure SiNW and the passivating layers taken as isolated. The results show a charge accumulation region around the Cl (Figure 5a) and H (Figure 5b) atoms, and a charge depletion around the Si atoms at the surface. This can be explained by the lower electronegativity of Si (1.9), with respect to H and Cl. The different shape of the differential charge density around H and Cl is related to the shape of the orbitals in the outer electronic shell, i.e., s- and p-orbitals for H and Cl, respectively. Quantitative analysis of charge transfer was performed using the Löwdin charge analysis, as implemented in Quantum Espresso. We calculated the partial charge on the clean SiNW, as well as the H- and Cl-SiNW. Our results show a negative charge transfer from the SiNW to the H and Cl layers of −0.07 and −0.27 e per adsorbed H and Cl atom, respectively. The lower charge transfer for H atoms is related to the lower electron affinity difference of H with respect to Si.

### 3.2. Octane Moiety Adsorbed on the (111) and (110) Surfaces of H- and Cl- SiNWs

We investigated the electronic properties of fully passivated H- and Cl- SiNWs with the octane moiety, C_8_H_17_, adsorbed on the (111) and (110) surfaces of the medium SiNW (from now indicated as Cl- and H-SiNWs-C_8_H_17_(111) and Cl- and H-SiNWs-C_8_H_17_(110), respectively). The choice of the octane moiety as adsorbed molecule was made because of its length, which well fits the objective to investigate the variation of some structural parameters such as rotation and/or alignment of the molecule with respect to the SiNW surfaces.

The supercell used for the adsorption cases is built by replicating the isolated medium SiNW twice along the longitudinal direction of the supercell. Therefore, each SiNW consists of 192 atoms of Si and 79 atoms of H or Cl (corresponding to the double size of the previous medium Cl- and H-SiNW). In the initial configuration, the octane moiety forms an angle, φ, of 60° with respect to the (111) or (110) plan, depending on the surface chosen to adsorb.

**Figure 6:**
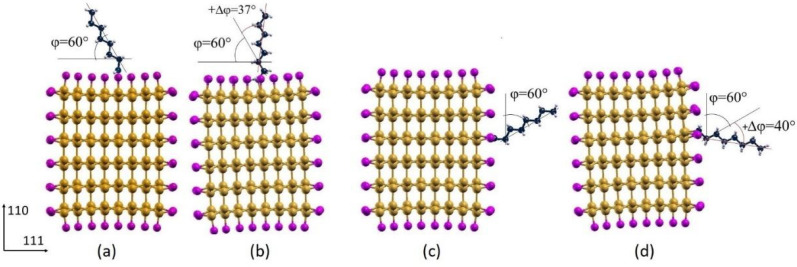
Initial (**a**,**c**) and optimized geometry (**b**,**d**) configurations of the octane moiety on the (111) (**a**,**b**) and (110) (**c**,**d**) surfaces of Cl-functionalized <112> oriented SiNWs (mustard color: Si atoms; magenta: Cl atoms; white: H atoms; blue: C atoms).

**Figure 7:**
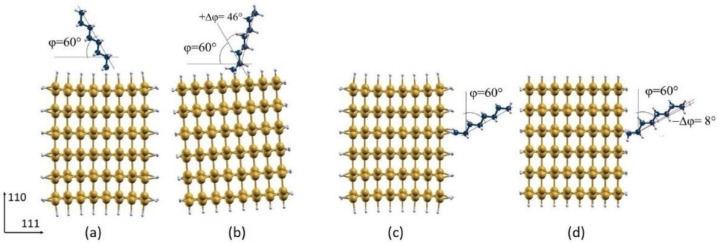
Initial (**a**,**c**) and optimized geometry (**b**,**d**) configurations of the octane moiety on the (111) (**a**,**b**) and (110) (**c**,**d**) surfaces of H-functionalized <112> oriented SiNWs (mustard color: Si atoms; white: H atoms; blue: C atoms).

After relaxation, φ increases for both the chlorinated configurations with an increment (+∆φ) of about 37° and 40° for the octane adsorbed on the (111) and (110) surface, respectively (Figure 6). A +∆φ of about 46° was observed for the hydrogenated system with the octane adsorbed on the (111) surface, while a decrease (−∆φ) of about 8° occurs for the hydrogenated system with the octane adsorption on the (110) surface (Figure 7).

The bond dissociation energy (BDE) of the modeled systems is calculated using the formula [31]:

(3) $$\mathrm{BDE}=(E_{X-SiNW}+E_{T})-E_{C8H17},$$

where $E_{X-SiNW}$ and $E_{C8H17}$ are the total energies of the relaxed X-SiWNs missing one X atom (X = H or Cl, respectively) in the binding site and the octane moiety, respectively. The results in Table 3 show a higher BDE value for both the chlorinated systems with respect to the hydrogenated systems, in agreement with the stability trend observed for the previous H- and Cl-SiNWs (Table 1). We found that the BDE of octane on Cl-SiNW is 1.00 eV larger than on H-SiNW, which can be attributed to the major electronic affinity of chlorine atoms relative to hydrogen atoms.

The formation energy per atom, *E_F_*, calculated according to (2), is quite independent on the adsorption surface. Indeed, we found *E_F_* (Cl-Si-C_8_H_17_)~−0.48 eV, and *E_F_* (H-Si-C_8_H_17_)~−0.053 eV. The Cl-Si-C_8_H_17_ presents the more stable configurations, with a small increase in the formation energy of about 0.03 eV with respect to the previous medium, Cl-SiNW. A stability improvement is found for the H-Si-C_8_H_17_ (111)/(110) with respect to the medium H-SiNW, with an *E_F_* decrease of about 0.02 eV.

**Figure 8:**
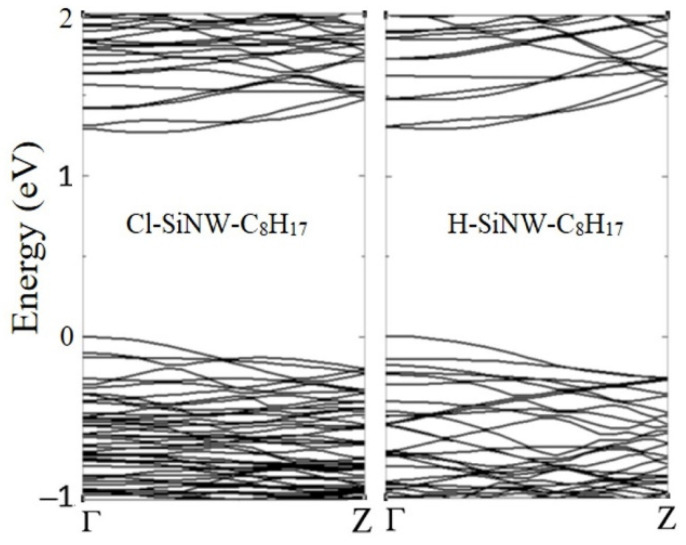
Band structure of Cl- (**left**) and H-SiNWS-C_8_H_17_ (**right**) systems. The top of the valence band is set to zero.

As was the case for the previously investigated systems (Figure 2), the band structure of the four Cl- and H- SiNW-C_8_H_17_ presents the maximum of the valence band near the Γ point, and the minimum of the conduction band near the Z point, exhibiting an indirect band gap in all cases (Figure 8). As the supercell size along the longitudinal direction is twice that of the isolated SiNW, the first Brillouin zone in reciprocal space shrinks and more energy dispersion curves result from the folding back at the boundaries, forming the so-called “bands folding”. The band structures and the PDOS show a weak dependence on the adsorption surface.

The resulting valence–conduction band gap is 1.26 eV and 1.29 eV for the chlorinated and hydrogenated systems, respectively. The comparison between the energy band gap of the medium Cl/H-SiNWs and the Cl/H-SiNW-C_8_H_17_ shows that *E_g_* (H-SiNW-C_8_H_17_) < *E_g_* (H-SiNW), and *E_g_* (Cl-SiNW-C_8_H_17_) > *E_g_* (Cl-SiNW), with a variation Δ*E_g_* of 0.01 and 0.07 eV, respectively. The variation in the energy gap of the Cl-SiNW, larger than for the H-SiNW, can be attributed to the substitution of the Cl atom in the adsorption site by less electronegative atoms.

**Figure 9:**
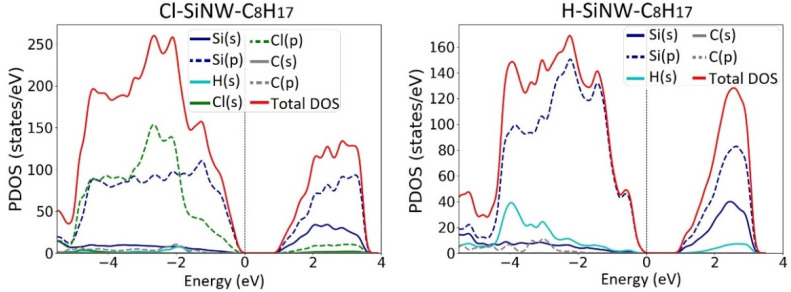
Projected electron density of states (PDOS) of Cl-SiNWS-C_8_H_17_ (**left**) and H-SiNWS-C_8_H_17_ (**right**) systems.

The PDOSs plotted in Figure 9 show that the aliphatic hydrocarbon contributes to the states enclosed in the valence and conduction bands, which are mainly composed of the silicon p-orbitals, Si(p). The octane moiety adsorption does not give rise to any energy level inside the energy band gap, differently from the case of alkene molecule adsorption, which introduces intragap energy levels [32].

#### Differential Charge Density of Cl- and H-SiNW-C_8_H_17_(111)/(110)

**Figure 10:**
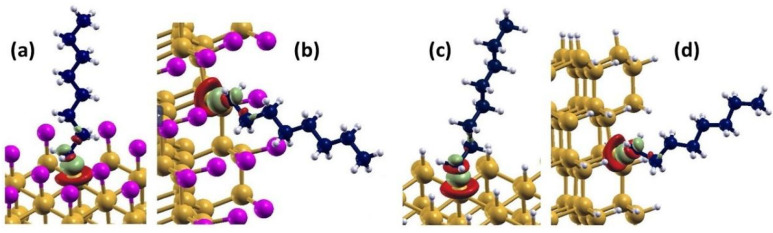
Isosurface of differential charge density of (**a**) Cl-SiNW-C_8_H_17_ and (**b**) H-SiNW-C_8_H_17_ with the adsorption of the octane moiety on (**a**,**c**) (111) surface, and (**b**,**d**) (110) surface. The isosurface value is set to 0.005 (mustard-color: Si atoms; white: H atoms; magenta: Cl atoms; blue: C atoms). Regions of green and red indicate charge accumulation and depletion, respectively.

The differential charge density was calculated to further investigate the electronic properties of Cl/H-SiNWs-C_8_H_17_(111)/(110). The differential charge density in this case has been calculated as the difference of the charge density of the full structure and the charge density of the passivated SiNW and alkane chain taken as isolated. Figure 10 shows the isosurface plots of the differential charge density for the four systems. The figures indicate that there is a charge accumulation between the Si and C atoms along the Si-C bond, and over the C atom close to the Si-C bond. Moreover, a charge depletion around the Si atom and near the bond edge of the C atom can be observed, as well as a more minor charge depletion and accumulation on the C-C bond edges close to the Si-C bond.

For all four adsorption cases investigated, the Löwdin charge analysis shows a partial charge of 48.5 e localized on the molecule, corresponding to an increase of 0.2 e with respect to the partial charge of 48.3 e of the isolated C_8_H_17_. Therefore, a charge transfer from the SiNW to the octane moiety is observed for both surface orientations with no dependence on Cl- or H-functionalization, which explains the increase in the stability.

## 4. Conclusions

Structures and energetics of two types of fully passivated SiNWs have been investigated using first-principles calculations based on the density functional theory. Two sets of three H- and Cl-passivated SiNWs with diameters of 0.7, 1.7 and 2.6 nm have been considered. The stability of the structures was investigated through the formation energy analysis. We found that, in terms of energy, the Cl atoms better passivate the dangling bonds of silicon, leading to a lower value of the formation energy. In both cases, due to the quantum confinement effect, the energy band gap decreases as the diameter of wire increases. However, the entity of this effect strongly depends on the passivation species and, in particular, it is influenced by the higher electronegativity of chlorine atoms. In all cases, the gap remains indirect according to the <112> wire orientation.

An octane moiety, C_8_H_17_, absorbed on the (111) and (110) surfaces of the two passivated medium diameter systems, has been modeled. This molecule was chosen because its length permits a better highlighting of their structural variations after relaxation, such as the inclination with respect to the surface of adsorption. In the unrelaxed configuration it forms an angle, φ, of 60° with respect to both the (111) and (110) H- and Cl-SiNW surfaces. After relaxation, φ varies sharply with respect to both surfaces of the chlorinated system, with an increment, +∆φ, of about 40°, while for the hydrogenated system we found a +∆φ of about 46°, with respect to the octane adsorption on the (110) surface, and a −∆φ of about 8° for the adsorption on the (111) surface. As far as we know, this work shows for the first time that the octane moiety adsorption on the surface of H- and Cl-passivated <211> SiNWs does not affect the electronic structure of the SiNWs. Indeed, the states localized on the molecules are embedded into the valence and conduction bands, with no generation of intragap energy levels. Therefore, the Si-C bond provided can enhance protection of the hydrogenated and chlorinated nanowire quantum surfaces against oxidation without substantial modification of the electronic properties. However, a charge transfer of 0.2 e from the SiNW to the alkane chain is found. This study paves the way for the use of alkane chains as passive molecules to effectively inhibit oxidation and extend the carrier lifetime.

## Figures and Tables

**Table 1:** Formation energy per atom of fully passivated H- Cl-functionalized <112> oriented SiNWs.

H-SiNWs	$E_{F}$ (eV/atom)	Cl-SiNWs	$E_{F}$ (eV/atom)
Si_24_H_20_	−0.0025	Si_24_Cl_20_	−0.73
Si_96_H_40_	−0.035	Si_96_Cl_40_	−0.51
Si_216_H_60_	−0.068	Si_216_Cl_60_	−0.41

**Table 2:** Energy band gap of fully passivated Cl- and H-functionalized <112> oriented SiNWs with different diameters.

H-SiNWs	*E_g_* (eV)	Cl-SiNWs	*E_g_* (eV)	*d* (nm)
Si_24_H_20_	2.36	Si_24_Cl_20_	2.04	0.7
Si_96_H_40_	1.30	Si_96_Cl_40_	1.19	1.7
Si_216_H_60_	0.98	Si_216_Cl_60_	0.95	2.6

**Table 3:** Bond dissociation energy values of the modelled systems.

NW Structure	BDE (kJ/Mol)
Cl-Si-C_8_H_17_ (111)	733.85
Cl-Si-C_8_H_17_ (110)	716.47
H-Si-C_8_H_17_ (111)	637.30
H-Si-C_8_H_17_ (110)	632.67

## Data Availability

The raw and processed data required to reproduce these findings will be provided upon reasonable request to anyone interested at any time.
